# Assessing financial protection in health: Does the choice of poverty line matter?

**DOI:** 10.1002/hec.4172

**Published:** 2020-10-02

**Authors:** John E. Ataguba

**Affiliations:** ^1^ Health Economics Unit, School of Public Health and Family Medicine, Health Sciences Faculty University of Cape Town Cape Town South Africa

**Keywords:** financial protection, impoverishment, out‐of‐pocket health spending, universal health coverage

## Abstract

Financial protection in health is an essential aspect of the universal health coverage discourse. It is about ensuring that paying for health services does not affect the ability of households and individuals to afford necessities. A well‐known way to assess financial protection is whether or not people are pushed into—or further into—poverty by paying out‐of‐pocket for health services. Although impoverishment from out‐of‐pocket health spending is not an explicit indicator of the sustainable development goals, it has gained prominence among researchers and policymakers because of its intuitive appeal and link to overall poverty reduction. Using data from Nigeria, this paper demonstrates that the choice of poverty line matters for assessing the impoverishing effect of paying out‐of‐pocket for health services. Among other things, the inconsistencies (or lack of dominance) could occur in ranking impoverishment levels by mutually exclusive groups within a country or in ranking different countries or a country over time. The implication is that the choice of poverty line could lead to manipulation of results for policy and for supporting an agenda that demonstrates an improvement in financial protection when this may not necessarily be the case.

## 1 INTRODUCTION

The progressive realization of universal health coverage (UHC) is acknowledged as relevant for many countries as they make significant efforts to reform their health systems to achieve the sustainable development goals (SDGs). One critical aspect for assessing progress towards UHC is understanding the level of financial protection that the health system offers (World Health Organization and World Bank, 2017). Traditionally, financial protection in health is assessed by looking at how health care payments, especially out‐of‐pocket health spending, make it difficult or impossible for individuals and households to demand basic household necessities like food, shelter, clothing, etc. (Wagstaff & van Doorslaer, 2003; Wagstaff, 2009). The two widely reported broad indicators of financial protection include financial catastrophe and impoverishment from paying out‐of‐pocket for health services. Pioneering papers that assessed financial catastrophe (Wagstaff & van Doorslaer, 2003; Xu et al., 2003) defined it as direct household expenditures on health services that exceed a fixed threshold of household income or expenditure (or any measure of capacity to pay). It has also been argued for the threshold to vary by household income or wealth status (Ataguba, 2012). Similarly, impoverishment from paying out‐of‐pocket for health services results from such payments that are sufficient to lower the living standards of individuals, pushing them below the poverty line (or in some cases, further into poverty for already poor individuals; Wagstaff & van Doorslaer, 2003). For impoverishment from out‐of‐pocket health spending, the main idea is that “no one ought to be pushed into poverty—or further into poverty—because of health care expenses” (Wagstaff & van Doorslaer, 2003, p. 927).

Unlike for the assessment of financial catastrophe, the indicators of impoverishment do not constitute official indicators of UHC within the broader SDGs framework. However, they link to the overarching goal of ending poverty as enshrined in the first SDG (World Health Organization and World Bank, 2017). In fact, it can be argued that to end poverty within and between countries, the link between the assessment of impoverishment from paying out‐of‐pocket for health services and the goal of attaining UHC is critical, especially in developing countries where poverty rate remains high (Ravallion & Chen, 2019). This is also essential because “health is an outcome, indicator and driver of sustainable development” (Webb, Small, & Gregor, 2019, p. 1).

While significant research on the theoretical, methodological and empirical aspects of assessing impoverishing out‐of‐pocket health spending exist, including how to measure out‐of‐pocket health spending (Lu, Chin, Lic, & Murray, 2009), especially in developing countries, the issues of consistency in impoverishment ranking (or some form of dominance) for various poverty lines within a country as well as comparisons between geographical locations (including countries) have received limited consideration. This remains the case even though cross‐country comparisons of these indicators exist (Wagstaff et al., 2015, 2018) and it is known that the correlation between households being both impoverished and incurring financial catastrophe is “sensitive to the choice of the poverty line” (Wagstaff et al., 2018, p. e191). In fact, the issues of poverty ordering and dominance have received significant attention in the traditional poverty literature (Davidson & Duclos, 2000; Duclos & Makdissi, 2005; Sahn & Stifel, 2000) but not for assessing impoverishment due to out‐of‐pocket health spending. Specifically, this paper makes the argument that the choice of poverty line matters when the analyst is interested in comparing or ranking impoverishment levels from paying out‐of‐pocket for health services by poverty lines in a country. It also shows potential inconsistencies or lack of dominance in comparing or ranking impoverishment levels by mutually exclusive groupings within a country (e.g., urban/rural, provinces, counties, states, etc.) or between countries or geographical locations such as cross‐country comparison of impoverishment from out‐of‐pocket health spending.

### 1.1 Conceptual framework

Consider an individual *i* with income before out‐of‐pocket health spending denoted as $y_{i}^{pre}$ and out‐of‐pocket spending on health in the same period as $x_{i}$. We denote individual *i*'s income after out‐of‐pocket health spending as $y_{i}^{post}=y_{i}^{pre}-x_{i}$ (i.e., income after paying out‐of‐pocket for health services). If the poverty line is denoted as PL, then the poverty indicator $P_{i}^{pre}$, is such that:

$$P_{i}^{pre}=\{\begin{array}{c} 1\\ 0 \end{array}\;\;\;\begin{array}{c} \text{if}\;\;y_{i}^{pre}<PL\\ \text{otherwise}\;\;\;\;\; \end{array}.\;\text{Similarly},\;P_{i}^{post}=\{\begin{array}{c} 1\\ 0 \end{array}\;\;\;\begin{array}{c} \text{if}\;\;y_{i}^{post}<PL\\ \text{otherwise}\;\;\;\;\;\; \end{array}.$$

Following Wagstaff and van Doorslaer (2003), the poverty headcounts before $\left(H^{pre}\right)$ and after $\left(H^{post}\right)$ out‐of‐pocket health spending are given as $H^{pre}=\frac{\sum_{i=1}^{N}P_{i}^{pre}}{N}$ and $H^{post}=\frac{\sum_{i=1}^{N}P_{i}^{post}}{N}$, respectively, where *N* is the sample or population size.

For simplicity, the impoverishing effect (in this case headcount, ΔH) of out‐of‐pocket health spending is obtained as:

(1) $$\Delta H_{PL}=H_{PL}^{post}-H_{PL}^{pre}\;\text{for}\;\text{each}\;\text{poverty}\;\text{line}\;\left(PL\right)$$

where $\Delta H_{PL}\geq 0\forall \;PL\in {\mathbb{R}}_{+}$

Based on the Foster‐Greer‐Thorbecke class of poverty (Foster, Greer, & Thorbecke, 1984), similar expressions can be written for the poverty gap, normalized poverty gap and the normalized mean poverty gap, etc. (Wagstaff & van Doorslaer, 2003). This paper uses the impoverishment headcount for illustration. However, the same argument can be extended to other measures of poverty, say the impoverishment gap.

For example, if we define the normalized poverty gap before and after out‐of‐pocket health spending, as $NG^{pre}=\frac{\left(\sum_{i=1}^{N}\frac{P_{i}^{pre}\left(PL-y_{i}^{pre}\right)}{PL}\right)}{N}\;$ and $NG^{post}=\frac{\left(\sum_{i=1}^{N}\frac{P_{i}^{post}\left(PL-y_{i}^{post}\right)}{PL}\right)}{N}$, respectively, then the effect of out‐of‐pocket health spending on the normalized poverty gap is given as:

(2) $$\Delta NG_{PL}=NG_{PL}^{post}-NG_{PL}^{pre}\;\text{for}\;\text{each}\;PL$$

**The issue:** *Within countries, the choice of poverty line matters for the ranking of impoverishment levels from paying out‐of‐pocket for health services*

**FIGURE 1:**
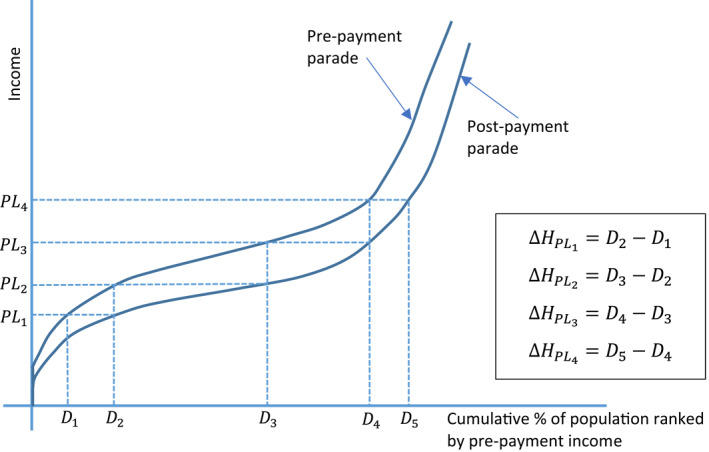
The implications of different poverty lines on the impoverishing impact (i.e., headcount) of out‐of‐pocket health spending using the Pen's Parade. *Source*: Based on the original illustration in Wagstaff and van Doorslaer (2003) [Colour figure can be viewed at wileyonlinelibrary.com]

The magnitude of impoverishment from out‐of‐pocket health spending, $\Delta H_{PL}$, will not vary monotonously with the choice of the poverty line
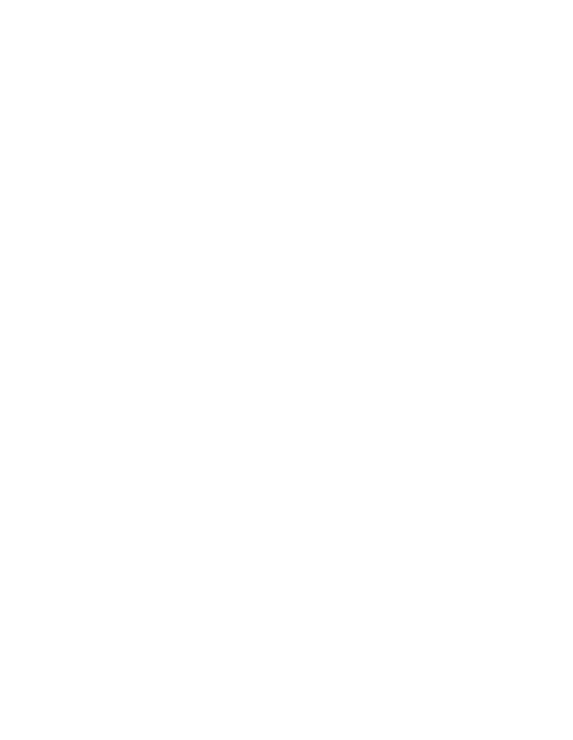
. For a given set of poverty lines, say, $PL_{1}<PL_{2}<\cdots <PL_{n}$, the ranking of corresponding estimates of impoverishing effects of out‐of‐pocket health spending ($\Delta H_{PL}$) does not follow any predetermined order. For example, if $\Delta H_{PL_{n}}>\Delta H_{PL_{n-1}}$, it may be the case that $\Delta H_{PL_{n-1}}$⪑$\Delta H_{PL_{n-2}}$, and so on. As shown in Figure 1, $\Delta H_{PL_{1}}<\Delta H_{PL_{2}}$ while $\Delta H_{PL_{2}}>\Delta H_{PL_{3}}.$

Also, an inconsistent ranking may arise when comparing impoverishment indicators in a country over time as it is difficult to ascertain whether the change in impoverishment observed over time is due to changes in the underlying income distribution, an improvement in financial protection or the inconsistency caused by choice of the poverty line noted above.

An extension can be deduced from how the ranking of impoverishment levels responds to the choice of poverty lines, especially when comparing two geographic areas, including groups within a country.

**Extension to the issue:** *The choice of poverty line matters for comparing and ranking impoverishment levels for mutually exclusive groupings, including between geographic locations*

**FIGURE 2:**
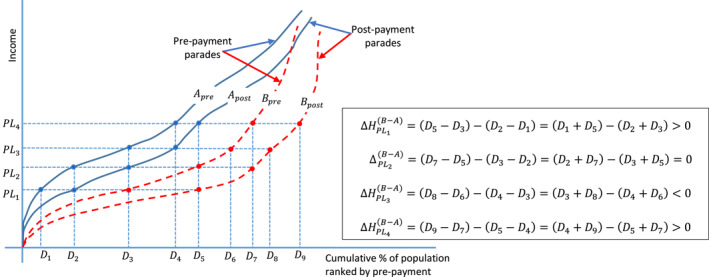
The implications of different poverty lines on changes in the impoverishing impact of out‐of‐pocket health spending between two groups using the Pen's Parade [Colour figure can be viewed at wileyonlinelibrary.com]

Let us define a set of mutually exclusive groups $G=\left\{g_{1},\ldots ,g_{n}\right\}$, where n≥2. Examples of mutually exclusive groups within a country include urban/rural, male‐ or female‐headed households, and so on. Between geographic locations also include between countries. Let $\Delta H_{PL_{1}}^{g_{1}}$ and $\Delta H_{PL_{1}}^{g_{2}}$ respectively represent the impoverishing effect of out‐of‐pocket health spending in groups 1 and 2 at the poverty line $PL_{1}$. If $\Delta H_{PL_{1}}^{g_{1}}>\Delta H_{PL_{1}}^{g_{2}}$, it may be the case that $\Delta H_{PL_{n}}^{g_{1}}>/\Delta H_{PL_{n}}^{g_{2}}\forall \;n$. For simplicity, this can be inferred from the fact that there is no monotonic relationship between the poverty lines and the impoverishment impact of out‐of‐pocket health spending. In order words, the impoverishing effect of out‐of‐pocket health spending may be higher in one group than the other at a specific poverty line but not at another. For example, as shown in Figure 2 for mutually exclusive groups *A* and *B*, the impoverishing effect of out‐of‐pocket health spending is higher in group *B* compared to group *A* at poverty lines $PL_{1}\left(\Delta H_{PL_{1}}^{\left(B-A\right)}>0\right)$ and $PL_{4}\left(\Delta H_{PL_{4}}^{\left(B-A\right)}>0\right)$ but this is not the case at $PL_{2}$ and $PL_{3}$ where the changes in the impoverishing effect of out‐of‐pocket health spending between groups *A* and *B* were zero $\left(\Delta H_{PL_{2}}^{\left(B-A\right)}=0\right)$ and negative $\left(\Delta H_{PL_{3}}^{\left(B-A\right)}<0\right)$, respectively. Further, this inconsistency or lack of dominance makes it difficult to compare or rank geographic locations, including countries by impoverishing effect of out‐of‐pocket spending even using the same international poverty line (e.g., $1.9/day or $3.2/day at 2011 purchasing power parity [PPP; World Health Organization and World Bank, 2017]). The inconsistencies in ranking groups could result from differences in the underlying income distributions between groups before out‐of‐pocket health spending ($y^{pre}$). However, it is critical to understand the impact of choosing one poverty line over another.

## 2 THE CASE OF NIGERIA

Nigeria is a lower‐middle‐income country, the most populous country in sub‐Saharan Africa and one of Africa's largest economies. The country is divided up into 36 states and one autonomous federal capital territory, Abuja. These states are further grouped into six geo‐political zones (North‐east, North‐west, North‐central, South‐east, South‐west and South‐south). The country has one of the largest shares of household out‐of‐pocket health spending in current health expenditure in the world. This share increased from 71% in 2013 to 77% in 2017 (World Health Organization, 2020). Also, over the past two decades, domestic general government health expenditure accounted for less than 20% of current health expenditure in the country (World Health Organization, 2020). Health insurance in the country is still under‐developed with only less than 5% of the population, predominantly federal government civil servants covered by the National Health Insurance Scheme established in 2005 (Uzochukwu et al., 2015). The very high share of household out‐of‐pocket health spending means that a substantial proportion of the population will be predisposed to financial catastrophe and impoverishment, making Nigeria an exciting country for empirical application.

This paper uses data from the 2008/2009 nationally representative Harmonized Nigeria National Living Standard Survey (HNLSS) conducted by the National Bureau of Statistics. Full details of the HNLSS is contained elsewhere (National Bureau of Statistics, 2010). The dataset with household expenditure contains information on about 35,000 households. Per capita household consumption (annualized) is used as a proxy for household income. Out‐of‐pocket health spending (computed per capita) comprises direct payments made by households at the point of using health services (public and private), which are not reimbursed by any prepayment scheme. Ataguba, Ichoku, Nwosu, and Akazili (2019) provide a detailed explanation of how to extract out‐of‐pocket health spending from the 2008/2009 HNLSS. Specifically, direct expenditures reported for consultations (outpatient health service use), hospital admission costs (inpatient health care costs), medicines and supplies related to admissions and outpatient services, drugs, and maternal and child health services are added up and annualized for each household.

The paper uses the $1.9/day (2011 PPP) and the $3.1/day (2011 PPP) as the lower‐ and upper‐bound international poverty lines, respectively. Stata is used to perform all estimations and graphing (StataCorp, 2017).

## 3 RESULTS AND DISCUSSION

**FIGURE 3:**
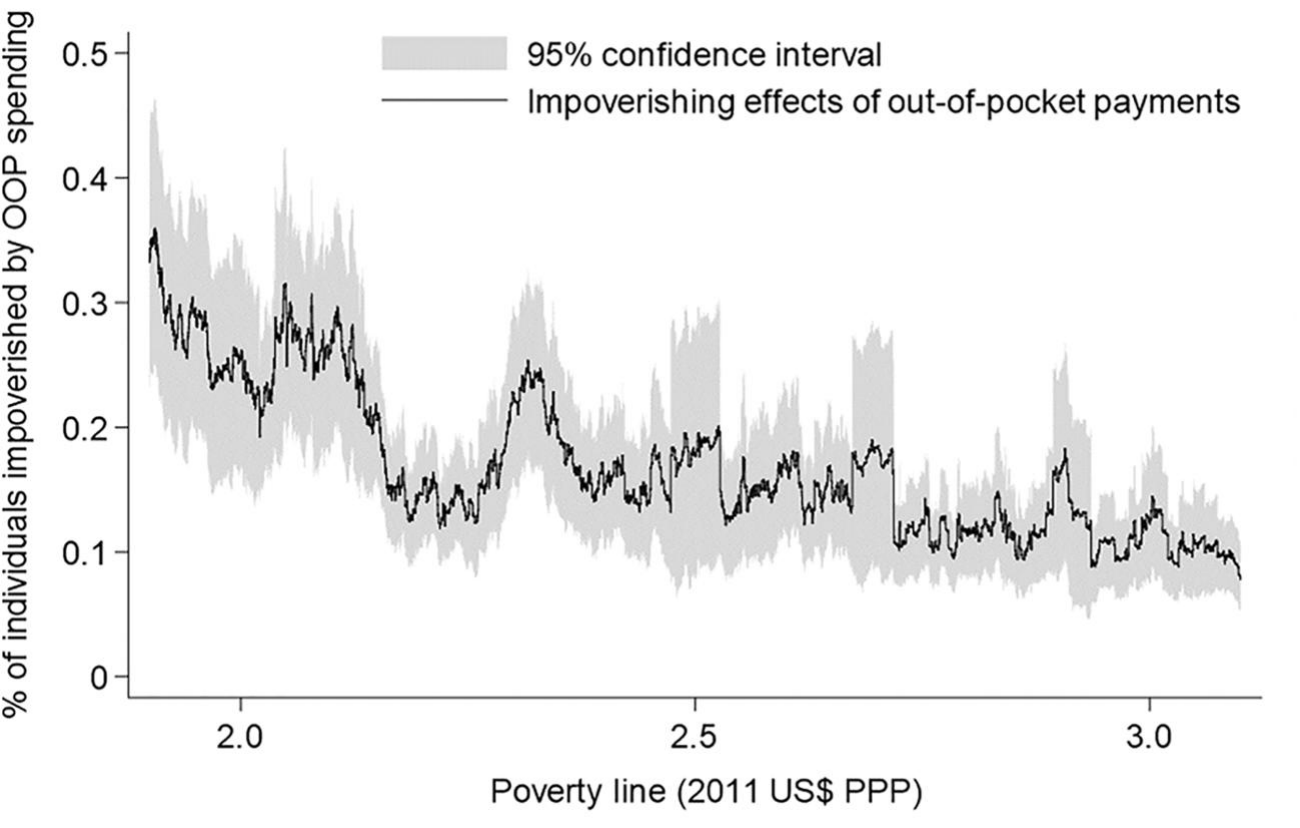
The effect of poverty lines on the assessment of the impoverishing impact of paying out‐of‐pocket (OOP) for health services in Nigeria, 2008/2009. See also Table S1 that shows the impoverishing effects for selected poverty lines. *Source*: Author's computation

The results in Figure 3 indicate that the choice of poverty lines affect the impoverishment headcount resulting from out‐of‐pocket health spending in Nigeria. The impoverishment headcounts are statistically significant and greater than zero for all the poverty lines. A general decline in the impoverishment headcount is reported by increasing the poverty line gradually from $1.9 to $3.1 per day, although this is not consistent. For example, the impoverishment headcount at the $2.2/day poverty line (0.147%) is lower than the headcount at the $2.3/day poverty line (0.219%). At the $2.4/day poverty line, the impoverishment headcount decreased to 0.163%. With a population of over 150 million people, it is estimated that over 220,000 people, 330,000 people and 240,000 people are impoverished by out‐of‐pocket health spending in Nigeria at poverty lines $2.2/day, $2.3/day and $2.4/day, respectively. If the $2.3/day poverty line is selected as opposed to the $2.2/day poverty line, an additional 110,000 people, which is non‐trivial, will be categorized as impoverished by paying out‐of‐pocket for health services in Nigeria. However, this difference will drop to about 20,000 people if the poverty line is set at $2.4/day instead of $2.2/day.

**FIGURE 4:**
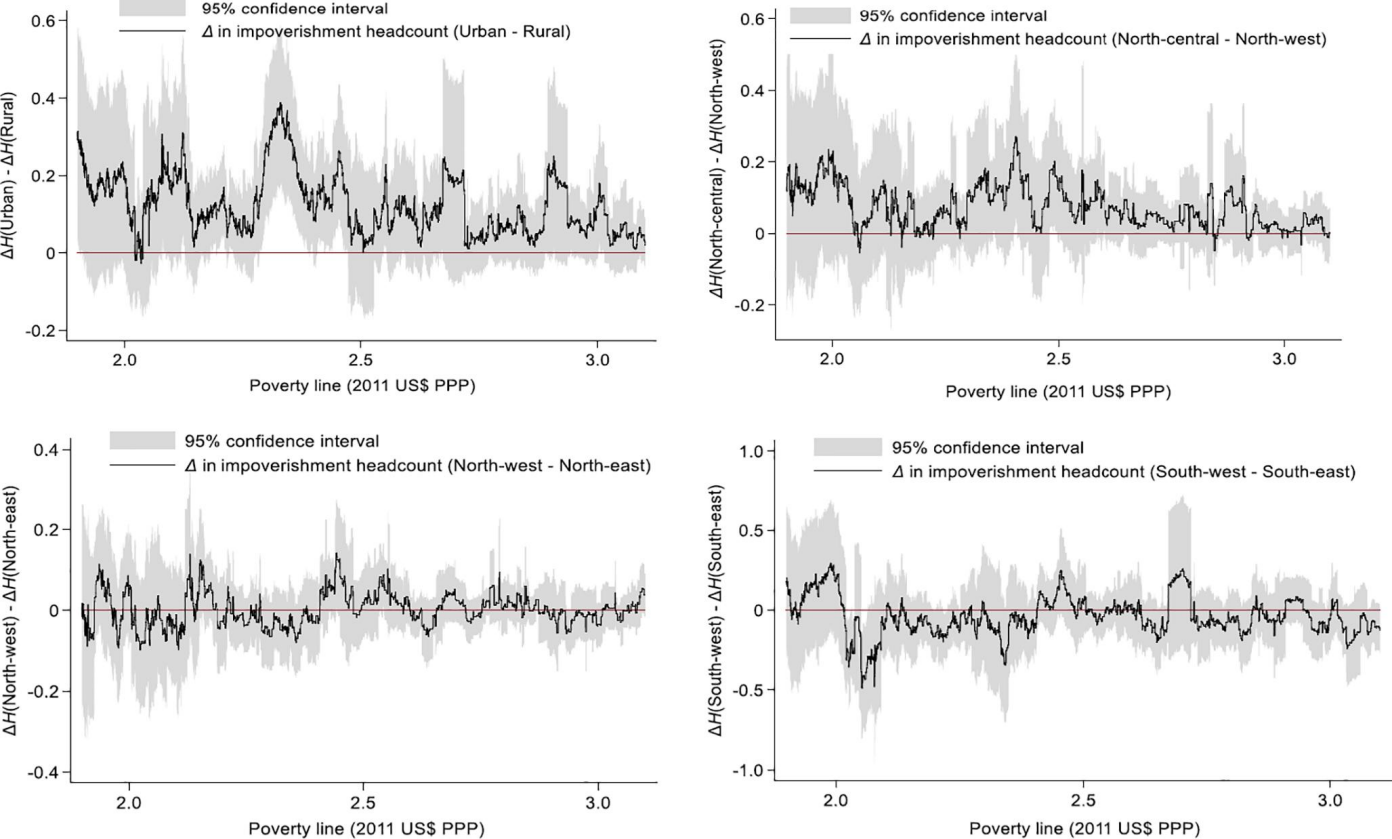
The effect of poverty lines on the assessment of impoverishment headcount from out‐of‐pocket health spending between selected groups in Nigeria, 2008/2009. As an example, ΔH(urban)−ΔH(rural) represents the difference in the impoverishment headcount due to out‐of‐pocket health spending between urban and rural areas. If ΔH(urban)−ΔH(rural)>0, impoverishment headcount is higher in the urban than in the rural area. See also Tables S2–S5 that shows the impoverishing effects for selected poverty lines. *Source*: Author's computation

In Figure 4, the effects of changing the poverty line on the assessment of impoverishment from out‐of‐pocket health spending by groups also indicate that the choice of the poverty line may matter. The zero lines in Figure 4 are used to assess if there are no statistically significant differences between the impoverishment headcounts of two mutually exclusive groups at some poverty lines in the case of Nigeria using the selected groups. In some cases, as shown in Figure 4, the differences are not statistically significant at the 5% level. For example, at the $2.5/day poverty line, there is no statistically significant difference between the impoverishment headcount in the rural and urban areas in Nigeria. At the $2.3/day or $2.4/day poverty lines, however, impoverishment headcounts are higher in the urban area than in the rural area. The results by geopolitical zones in Nigeria also show that, depending on the choice of the poverty line, impoverishment headcount may be different (i.e., where there exists a statistically significant difference in the impoverishment headcounts between zones) or the same between two zones (i.e., a case where the difference in impoverishment headcounts between zones is not statistically different from zero). The impoverishment headcount could be higher in one zone than in another and this could switch with another very close poverty line. For example, at the $2.2/day poverty line, the impoverishment from out‐of‐pocket health spending is significantly higher in the South‐east than the South‐west zone (Figure 4). However, at the $2.1/day and $2.3/day poverty lines, the impoverishment headcounts in both zones are not significantly different from each other. All things being equal, this implies that at the $2.2/day poverty line, the South‐west zone in Nigeria is performing better than the South‐east zone but at the $2.1/day and $2.3/day poverty lines, both geopolitical zones are identical in terms of the proportion of their population that is impoverished by out‐of‐pocket health spending.

Although only illustrated for a case within a country (e.g., by comparing different geopolitical zones in Nigeria), it can be deduced by extension that these results may point to potential inconsistencies in the ranking of geographical locations, including different countries by impoverishing effects of out‐of‐pocket health spending, for small changes in the international poverty lines. Also, even within a country, there could be inconsistency in assessing impoverishment from paying out‐of‐pocket for health services over time for small changes in the poverty line. While some of these inconsistencies may be due to differences in the underlying income distributions, the choice of a poverty line remains critical as different poverty lines lend themselves to different policy conclusions.

For brevity, this paper shows the results using the impoverishment headcount. The same analogy can be extended to other measures of impoverishment, including poverty intensity (i.e., gap) and severity. This has been introduced briefly in Equation (2) for the normalized poverty gap. In essence, it is about assessing the sensitivity of the impoverishing effect of out‐of‐pocket health spending, using any measure of poverty, to changes in poverty lines. This paper uses the headcount measure mainly because it is intuitive and widely reported in many health economics and public health journals and policy documents. In fact, this simplistic nature makes the headcount measure appealing to politicians and policymakers as opposed to the intensity and severity of impoverishment.

The goal of progressive realization of universal financial protection, within the UHC debate, means that countries must demonstrate an ongoing improvement in financial protection, for example, through a decline in the impoverishing impact of paying out‐of‐pocket for health services. Unfortunately, this becomes very challenging due to the potential inconsistencies and non‐dominance in the results. While other factors such as the way out‐of‐pocket health spending is computed (Lu et al., 2009) and the nature of income distribution may affect the assessment of financial protection in health, an improvement in financial protection may be demonstrated at one poverty line but a worsening or an unchanged situation at a very close poverty line. These potential inconsistencies may be exploited by policymakers and politicians to demonstrate “success” just be choosing the results based on a poverty line that aligns with their political agenda. Even when this is not the case, it may well be that the results from using the popular poverty lines (e.g., $1.9 or $3.2/day) back their political agenda while conflicting results may come from very close poverty lines. The question then is: what is the best choice of the poverty line for assessing impoverishment resulting from out‐of‐pocket health spending? Even though a lower poverty line is recommended for poorer countries and a higher poverty line for richer countries, as this paper shows, the answer to this question is not very obvious as the choice of poverty line matters when assessing the poverty implications of paying out‐of‐pocket for health services. This could exist both within and between countries, and over time.

## 4 CONCLUSION

Financial protection has gained prominence internationally, especially among researchers and policymakers. One way to assess financial protection in health is the extent to which households are protected from being impoverished or pushed further into poverty because of the need to pay for health services out‐of‐pocket. Apart from the impact of any differences in the distribution of income or how out‐of‐pocket health spending is computed, this paper argues that the assessment of impoverishment from paying out‐of‐pocket for health services and the conclusions that may arise can be sensitive to the choice of poverty lines, especially for comparisons in a country over time, between groups in a country and for cross‐country comparison. This means that policymakers and politicians may manipulate the results to suit their political agenda. As countries aim to achieve UHC and to ensure financial protection for their population, this paper highlights the importance of assessing the implications of different poverty lines for the ordering or ranking of impoverishment indicators.

## Supporting information

Supporting Information 1

## References

[hec4172-bib-0001] Ataguba, J. E. (2012). Reassessing catastrophic health care payments with a Nigerian case study. Health Economics, Policy and Law, 7, 309–326. 10.1017/S1744133110000356 21310095

[hec4172-bib-0002] Ataguba, J. E. , Ichoku, H. E. , Nwosu, C. O. , & Akazili, J. (2019). An alternative approach to decomposing the redistributive effect of health financing between and within groups using the gini index: The case of out‐of‐pocket payments in Nigeria. Applied Health Economics and Health Policy, 2019, 1–11. 10.1007/s40258-019-00520-4 PMC7716861 31628664

[hec4172-bib-0003] Davidson, R. , & Duclos, J.‐Y. (2000). Statistical inference for stochastic dominance and for the measurement of poverty and inequality. Econometrica, 68, 1435–1464.

[hec4172-bib-0004] Duclos, J. Y. , & Makdissi, P. (2005). Sequential stochastic dominance and the robustness of poverty orderings. Review of Income and Wealth, 51, 63–87.

[hec4172-bib-0005] Foster, J. , Greer, J. , & Thorbecke, E. (1984). A class of decomposable poverty measures. Econometrica, 52, 761–766.

[hec4172-bib-0006] Lu, C. , Chin, B. , Lic, G. , & Murray, C. J. L. (2009). Limitations of methods for measuring out‐of‐pocket and catastrophic private health expenditures. Bulletin of the World Health Organization, 87, 238–244. 19377721 10.2471/BLT.08.054379 PMC2654642

[hec4172-bib-0007] National Bureau of Statistics . (2010). Harmonized Nigeria living standard Survey (HNLSS) 2008/2009. Abuja, Nigeria: National Bureau of Statistics.

[hec4172-bib-0008] Ravallion, M. , & Chen, S. (2019). Global poverty measurement when relative income matters. Journal of Public Economics, 177, 1–13.

[hec4172-bib-0009] Sahn, D. E. , & Stifel, D. C. (2000). Poverty comparisons over time and across countries in Africa. World Development, 28, 2123–2155.

[hec4172-bib-0010] StataCorp (2017). Stata: Release 15 ‐ statistical software. College Station, TX: StataCorp LP.

[hec4172-bib-0011] Uzochukwu, B. , Ughasoro, M. , Etiaba, E. , Okwuosa, C. , Envuladu, E. , & Onwujekwe, O. (2015). Health care financing in Nigeria: Implications for achieving universal health coverage. Nigerian Journal of Clinical Practice, 18, 437–444. 25966712 10.4103/1119-3077.154196

[hec4172-bib-0012] Wagstaff, A. (2009). Measuring financial protection in health In Smith P. C., Mossialos E., Papanicolas I., & Leatherman S. (Eds.), Performance measurement for health system improvement: Experiences, challenges and prospects. New York, NY: Cambridge University Press.

[hec4172-bib-0013] Wagstaff, A. , Dmytraczenko, T. , Almeida, G. , Buisman, L. , Eozenou, P. H.‐V. , Bredenkamp, C. , … Molina, S. (2015). Assessing Latin America's progress toward achieving universal health coverage. Health Affairs, 34, 1704–1712. 26438747 10.1377/hlthaff.2014.1453

[hec4172-bib-0014] Wagstaff, A. , Flores, G. , Smitz, M.‐F. , Hsu, J. , Chepynoga, K. , & Eozenou, P. (2018). Progress on impoverishing health spending in 122 countries: A retrospective observational study. The Lancet Global Health, 6, e180–e192. 29248366 10.1016/S2214-109X(17)30486-2

[hec4172-bib-0015] Wagstaff, A. , & van Doorslaer, E. (2003). Catastrophe and impoverishment in paying for health care: With applications to vietnam 1993‐98. Health Economics, 12, 921–933. 14601155 10.1002/hec.776

[hec4172-bib-0016] Webb, D. , Small, R. , & Gregor, E. (2019). Universal health coverage for sustainable development. New York, NY: United Nations Development Programme.

[hec4172-bib-0017] World Health Organization . (2020). Global health expenditure database [Online]. Geneva, Switzerland: World Health Organization Retrieved from https://apps.who.int/nha/database/ViewData/Indicators/en

[hec4172-bib-0018] World Health OrganizationWorld Bank . (2017). Tracking universal health coverage: 2017 global monitoring report. Geneva, Switzerland: World Health Organization and the World Bank.

[hec4172-bib-0019] Xu, K. , Evans, D. B. , Kawabata, K. , Zeramdini, R. , Klavus, J. , & Murray, C. J. L. (2003). Household catastrophic health expenditure: A multicountry analysis. The Lancet, 362, 111–117. 10.1016/S0140-6736(03)13861-5 12867110

