# Supplementary material for: Assessing financial protection in health: Does the choice of poverty line matter?
Source: Health Econ. 2020 Oct 2;30(1):186–93. doi: 10.1002/hec.4172 (PMC7756704; doi:10.1002/hec.4172)
Supplement: Supplementary file 1 — Supporting Information 1 [file HEC-30-186-s001.docx]

**Assessing financial protection in health: does the choice of poverty line matter?**

**Appendix 1**

Table 1: The effect of poverty lines on the assessment of the impoverishing impact of paying out-of-pocket for health services in Nigeria, 2008/09

| **Poverty line (US$)** | **Impoverishment headcount (%)** | **Standard error** | **T-statistic** |
| --- | --- | --- | --- |
| 1.90 | 0.3348 | 0.0530 | 6.3127 |
| 1.91 | 0.3507 | 0.0530 | 6.6221 |
| 1.92 | 0.2895 | 0.0481 | 6.0129 |
| 1.93 | 0.2690 | 0.0454 | 5.9239 |
| 1.94 | 0.2646 | 0.0455 | 5.8125 |
| 1.95 | 0.2927 | 0.0472 | 6.2007 |
| 1.96 | 0.2870 | 0.0472 | 6.0845 |
| 1.97 | 0.2318 | 0.0441 | 5.2543 |
| 1.98 | 0.2409 | 0.0445 | 5.4171 |
| 1.99 | 0.2438 | 0.0452 | 5.3940 |
| 2.00 | 0.2571 | 0.0468 | 5.4952 |
| 2.01 | 0.2435 | 0.0434 | 5.6155 |
| 2.02 | 0.2258 | 0.0417 | 5.4120 |
| 2.03 | 0.2292 | 0.0333 | 6.8862 |
| 2.04 | 0.2477 | 0.0357 | 6.9313 |
| 2.05 | 0.3129 | 0.0558 | 5.6048 |
| 2.06 | 0.2728 | 0.0419 | 6.5169 |
| 2.07 | 0.2561 | 0.0405 | 6.3261 |
| 2.08 | 0.2968 | 0.0471 | 6.2990 |
| 2.09 | 0.2617 | 0.0434 | 6.0247 |
| 2.10 | 0.2600 | 0.0413 | 6.2952 |
| 2.11 | 0.2905 | 0.0454 | 6.4041 |
| 2.12 | 0.2396 | 0.0427 | 5.6176 |
| 2.13 | 0.2374 | 0.0431 | 5.5021 |
| 2.14 | 0.2088 | 0.0320 | 6.5288 |
| 2.15 | 0.2118 | 0.0282 | 7.5152 |
| 2.16 | 0.1815 | 0.0232 | 7.8091 |
| 2.17 | 0.1491 | 0.0232 | 6.4188 |
| 2.18 | 0.1621 | 0.0251 | 6.4598 |
| 2.19 | 0.1279 | 0.0192 | 6.6660 |
| 2.20 | 0.1468 | 0.0221 | 6.6554 |
| 2.21 | 0.1518 | 0.0211 | 7.1860 |
| 2.22 | 0.1307 | 0.0185 | 7.0691 |
| 2.23 | 0.1310 | 0.0189 | 6.9313 |
| 2.24 | 0.1514 | 0.0212 | 7.1552 |
| 2.25 | 0.1256 | 0.0205 | 6.1302 |
| 2.26 | 0.1242 | 0.0224 | 5.5548 |
| 2.27 | 0.1468 | 0.0263 | 5.5821 |
| 2.28 | 0.1730 | 0.0291 | 5.9454 |
| 2.29 | 0.1781 | 0.0279 | 6.3795 |
| 2.30 | 0.2193 | 0.0343 | 6.3848 |
| 2.31 | 0.2296 | 0.0360 | 6.3814 |
| 2.32 | 0.2403 | 0.0360 | 6.6804 |
| 2.33 | 0.2422 | 0.0371 | 6.5263 |
| 2.34 | 0.1968 | 0.0330 | 5.9637 |
| 2.35 | 0.2052 | 0.0345 | 5.9471 |
| 2.36 | 0.1744 | 0.0286 | 6.0959 |
| 2.37 | 0.1684 | 0.0261 | 6.4489 |
| 2.38 | 0.1515 | 0.0238 | 6.3579 |
| 2.39 | 0.1453 | 0.0240 | 6.0493 |
| 2.40 | 0.1632 | 0.0268 | 6.0806 |
| 2.41 | 0.1574 | 0.0258 | 6.0909 |
| 2.42 | 0.1631 | 0.0261 | 6.2478 |
| 2.43 | 0.1415 | 0.0238 | 5.9550 |
| 2.44 | 0.1354 | 0.0225 | 6.0278 |
| 2.45 | 0.1400 | 0.0266 | 5.2715 |
| 2.46 | 0.1752 | 0.0319 | 5.4969 |
| 2.47 | 0.1418 | 0.0287 | 4.9405 |
| 2.48 | 0.1744 | 0.0521 | 3.3435 |
| 2.49 | 0.1918 | 0.0521 | 3.6772 |
| 2.50 | 0.1788 | 0.0507 | 3.5239 |
| 2.51 | 0.1877 | 0.0511 | 3.6750 |
| 2.52 | 0.1852 | 0.0510 | 3.6343 |
| 2.53 | 0.1447 | 0.0256 | 5.6434 |
| 2.54 | 0.1286 | 0.0239 | 5.3818 |
| 2.55 | 0.1397 | 0.0250 | 5.5863 |
| 2.56 | 0.1440 | 0.0247 | 5.8326 |
| 2.57 | 0.1459 | 0.0272 | 5.3704 |
| 2.58 | 0.1557 | 0.0285 | 5.4558 |
| 2.59 | 0.1500 | 0.0268 | 5.6068 |
| 2.60 | 0.1728 | 0.0297 | 5.8122 |
| 2.61 | 0.1726 | 0.0294 | 5.8693 |
| 2.62 | 0.1336 | 0.0224 | 5.9529 |
| 2.63 | 0.1229 | 0.0184 | 6.6701 |
| 2.64 | 0.1353 | 0.0243 | 5.5689 |
| 2.65 | 0.1645 | 0.0266 | 6.1909 |
| 2.66 | 0.1417 | 0.0240 | 5.8955 |
| 2.67 | 0.1385 | 0.0238 | 5.8112 |
| 2.68 | 0.1675 | 0.0480 | 3.4881 |
| 2.69 | 0.1765 | 0.0486 | 3.6288 |
| 2.70 | 0.1830 | 0.0487 | 3.7574 |
| 2.71 | 0.1725 | 0.0483 | 3.5747 |
| 2.72 | 0.1077 | 0.0164 | 6.5767 |
| 2.73 | 0.1090 | 0.0167 | 6.5155 |
| 2.74 | 0.1200 | 0.0200 | 5.9993 |
| 2.75 | 0.1256 | 0.0200 | 6.2844 |
| 2.76 | 0.1045 | 0.0153 | 6.8268 |
| 2.77 | 0.1133 | 0.0168 | 6.7459 |
| 2.78 | 0.1027 | 0.0152 | 6.7632 |
| 2.79 | 0.1258 | 0.0227 | 5.5430 |
| 2.80 | 0.1134 | 0.0185 | 6.1274 |
| 2.81 | 0.1226 | 0.0201 | 6.1036 |
| 2.82 | 0.1254 | 0.0199 | 6.3019 |
| 2.83 | 0.1233 | 0.0204 | 6.0466 |
| 2.84 | 0.1175 | 0.0238 | 4.9357 |
| 2.85 | 0.1100 | 0.0188 | 5.8588 |
| 2.86 | 0.0976 | 0.0165 | 5.9334 |
| 2.87 | 0.1033 | 0.0183 | 5.6377 |
| 2.88 | 0.1123 | 0.0194 | 5.7851 |
| 2.89 | 0.1332 | 0.0252 | 5.2795 |
| 2.90 | 0.1565 | 0.0412 | 3.7995 |
| 2.91 | 0.1619 | 0.0409 | 3.9558 |
| 2.92 | 0.1309 | 0.0379 | 3.4521 |
| 2.93 | 0.1306 | 0.0380 | 3.4387 |
| 2.94 | 0.0895 | 0.0157 | 5.6955 |
| 2.95 | 0.1094 | 0.0191 | 5.7273 |
| 2.96 | 0.1101 | 0.0192 | 5.7362 |
| 2.97 | 0.0948 | 0.0162 | 5.8655 |
| 2.98 | 0.1123 | 0.0200 | 5.6151 |
| 2.99 | 0.1067 | 0.0180 | 5.9140 |
| 3.00 | 0.1249 | 0.0256 | 4.8739 |
| 3.01 | 0.1327 | 0.0268 | 4.9553 |
| 3.02 | 0.1135 | 0.0240 | 4.7319 |
| 3.03 | 0.0911 | 0.0151 | 6.0159 |
| 3.04 | 0.1007 | 0.0192 | 5.2353 |
| 3.05 | 0.1064 | 0.0218 | 4.8814 |
| 3.06 | 0.1123 | 0.0220 | 5.1128 |
| 3.07 | 0.1038 | 0.0190 | 5.4746 |
| 3.08 | 0.0990 | 0.0147 | 6.7440 |
| 3.09 | 0.0978 | 0.0155 | 6.2996 |
| 3.10 | 0.0779 | 0.0137 | 5.6656 |

*Note*: The results in this Table are shown using poverty lines with an interval of $0.01. However, the corresponding Figure in the main manuscript uses a detailed set of poverty lines with a narrower interval.

Table 2: The effect of poverty lines on the assessment of impoverishment headcount from out-of-pocket health spending between urban and rural areas in Nigeria, 2008/09

| **Poverty line (US$)** | **Change in impoverishment headcount (%)** | **Standard error** | **T-statistic** |
| --- | --- | --- | --- |
| 1.90 | 0.3075 | 0.1368 | 2.2487 |
| 1.91 | 0.2519 | 0.1340 | 1.8793 |
| 1.92 | 0.2163 | 0.1227 | 1.7623 |
| 1.93 | 0.1736 | 0.1173 | 1.4796 |
| 1.94 | 0.1341 | 0.1181 | 1.1353 |
| 1.95 | 0.1838 | 0.1226 | 1.4991 |
| 1.96 | 0.1684 | 0.1222 | 1.3785 |
| 1.97 | 0.1486 | 0.1155 | 1.2862 |
| 1.98 | 0.2087 | 0.1175 | 1.7759 |
| 1.99 | 0.1985 | 0.1188 | 1.6705 |
| 2.00 | 0.2136 | 0.1220 | 1.7512 |
| 2.01 | 0.1133 | 0.1096 | 1.0340 |
| 2.02 | 0.1042 | 0.1068 | 0.9755 |
| 2.03 | 0.0494 | 0.0743 | 0.6648 |
| 2.04 | -0.0150 | 0.0751 | -0.1994 |
| 2.05 | 0.1469 | 0.1415 | 1.0376 |
| 2.06 | 0.1368 | 0.1008 | 1.3575 |
| 2.07 | 0.1348 | 0.0967 | 1.3932 |
| 2.08 | 0.2435 | 0.1182 | 2.0608 |
| 2.09 | 0.2037 | 0.1120 | 1.8181 |
| 2.10 | 0.1572 | 0.1037 | 1.5149 |
| 2.11 | 0.2257 | 0.1162 | 1.9420 |
| 2.12 | 0.1938 | 0.1102 | 1.7591 |
| 2.13 | 0.1896 | 0.1118 | 1.6954 |
| 2.14 | 0.0972 | 0.0769 | 1.2640 |
| 2.15 | 0.0457 | 0.0629 | 0.7275 |
| 2.16 | 0.0837 | 0.0548 | 1.5275 |
| 2.17 | 0.0823 | 0.0523 | 1.5729 |
| 2.18 | 0.1053 | 0.0569 | 1.8505 |
| 2.19 | 0.1206 | 0.0479 | 2.5184 |
| 2.20 | 0.1114 | 0.0512 | 2.1770 |
| 2.21 | 0.1397 | 0.0524 | 2.6654 |
| 2.22 | 0.0397 | 0.0406 | 0.9763 |
| 2.23 | 0.1025 | 0.0470 | 2.1825 |
| 2.24 | 0.0671 | 0.0494 | 1.3571 |
| 2.25 | 0.0437 | 0.0487 | 0.8977 |
| 2.26 | 0.0510 | 0.0547 | 0.9312 |
| 2.27 | 0.0400 | 0.0600 | 0.6664 |
| 2.28 | 0.1424 | 0.0745 | 1.9098 |
| 2.29 | 0.1505 | 0.0712 | 2.1153 |
| 2.30 | 0.2614 | 0.0904 | 2.8903 |
| 2.31 | 0.3029 | 0.0954 | 3.1753 |
| 2.32 | 0.3248 | 0.0963 | 3.3730 |
| 2.33 | 0.3566 | 0.1006 | 3.5458 |
| 2.34 | 0.3183 | 0.0898 | 3.5447 |
| 2.35 | 0.3371 | 0.0937 | 3.5978 |
| 2.36 | 0.2524 | 0.0767 | 3.2907 |
| 2.37 | 0.2039 | 0.0687 | 2.9681 |
| 2.38 | 0.1540 | 0.0604 | 2.5482 |
| 2.39 | 0.1192 | 0.0599 | 1.9908 |
| 2.40 | 0.1371 | 0.0671 | 2.0434 |
| 2.41 | 0.1504 | 0.0656 | 2.2930 |
| 2.42 | 0.1610 | 0.0664 | 2.4246 |
| 2.43 | 0.1397 | 0.0599 | 2.3303 |
| 2.44 | 0.1104 | 0.0558 | 1.9780 |
| 2.45 | 0.2037 | 0.0713 | 2.8589 |
| 2.46 | 0.2288 | 0.0853 | 2.6815 |
| 2.47 | 0.1615 | 0.0764 | 2.1136 |
| 2.48 | 0.0679 | 0.1010 | 0.6725 |
| 2.49 | 0.1099 | 0.1010 | 1.0883 |
| 2.50 | 0.0481 | 0.0942 | 0.5106 |
| 2.51 | 0.0265 | 0.0936 | 0.2837 |
| 2.52 | 0.0401 | 0.0936 | 0.4280 |
| 2.53 | 0.1007 | 0.0635 | 1.5874 |
| 2.54 | 0.0635 | 0.0591 | 1.0738 |
| 2.55 | 0.1479 | 0.0650 | 2.2763 |
| 2.56 | 0.0531 | 0.0605 | 0.8788 |
| 2.57 | 0.0897 | 0.0655 | 1.3678 |
| 2.58 | 0.1142 | 0.0700 | 1.6318 |
| 2.59 | 0.1454 | 0.0686 | 2.1199 |
| 2.60 | 0.1058 | 0.0718 | 1.4735 |
| 2.61 | 0.0926 | 0.0713 | 1.2998 |
| 2.62 | 0.0600 | 0.0515 | 1.1670 |
| 2.63 | 0.0475 | 0.0422 | 1.1243 |
| 2.64 | 0.0809 | 0.0611 | 1.3251 |
| 2.65 | 0.1053 | 0.0662 | 1.5917 |
| 2.66 | 0.0938 | 0.0607 | 1.5443 |
| 2.67 | 0.1059 | 0.0610 | 1.7370 |
| 2.68 | 0.2159 | 0.1316 | 1.6411 |
| 2.69 | 0.1953 | 0.1322 | 1.4766 |
| 2.70 | 0.1859 | 0.1321 | 1.4079 |
| 2.71 | 0.1830 | 0.1311 | 1.3951 |
| 2.72 | 0.0238 | 0.0360 | 0.6608 |
| 2.73 | 0.0224 | 0.0366 | 0.6138 |
| 2.74 | 0.0403 | 0.0473 | 0.8529 |
| 2.75 | 0.0419 | 0.0471 | 0.8881 |
| 2.76 | 0.0268 | 0.0349 | 0.7674 |
| 2.77 | 0.0646 | 0.0407 | 1.5862 |
| 2.78 | 0.0490 | 0.0361 | 1.3574 |
| 2.79 | 0.0833 | 0.0587 | 1.4183 |
| 2.80 | 0.0550 | 0.0454 | 1.2132 |
| 2.81 | 0.0853 | 0.0508 | 1.6787 |
| 2.82 | 0.0567 | 0.0484 | 1.1721 |
| 2.83 | 0.0763 | 0.0507 | 1.5068 |
| 2.84 | 0.0825 | 0.0616 | 1.3384 |
| 2.85 | 0.0644 | 0.0476 | 1.3540 |
| 2.86 | 0.0460 | 0.0393 | 1.1719 |
| 2.87 | 0.0493 | 0.0455 | 1.0845 |
| 2.88 | 0.0519 | 0.0474 | 1.0965 |
| 2.89 | 0.1300 | 0.0660 | 1.9687 |
| 2.90 | 0.1993 | 0.1124 | 1.7730 |
| 2.91 | 0.2003 | 0.1115 | 1.7970 |
| 2.92 | 0.1873 | 0.1039 | 1.8019 |
| 2.93 | 0.1822 | 0.1039 | 1.7528 |
| 2.94 | 0.0700 | 0.0400 | 1.7486 |
| 2.95 | 0.0756 | 0.0456 | 1.6574 |
| 2.96 | 0.0779 | 0.0461 | 1.6882 |
| 2.97 | 0.0593 | 0.0372 | 1.5959 |
| 2.98 | 0.0718 | 0.0490 | 1.4646 |
| 2.99 | 0.0607 | 0.0460 | 1.3191 |
| 3.00 | 0.1216 | 0.0684 | 1.7776 |
| 3.01 | 0.1475 | 0.0712 | 2.0705 |
| 3.02 | 0.0979 | 0.0631 | 1.5512 |
| 3.03 | 0.0232 | 0.0341 | 0.6811 |
| 3.04 | 0.0437 | 0.0474 | 0.9217 |
| 3.05 | 0.0597 | 0.0557 | 1.0713 |
| 3.06 | 0.0775 | 0.0565 | 1.3706 |
| 3.07 | 0.0503 | 0.0463 | 1.0865 |
| 3.08 | 0.0119 | 0.0317 | 0.3738 |
| 3.09 | 0.0525 | 0.0358 | 1.4664 |
| 3.10 | 0.0199 | 0.0302 | 0.6597 |

*Note*: The results in this Table are shown using poverty lines with an interval of $0.01. However, the corresponding Figure in the main manuscript uses a detailed set of poverty lines with a narrower interval.

Table 3: The effect of poverty lines on the assessment of impoverishment headcount from out-of-pocket health spending between north central and north west regions in Nigeria, 2008/09

| **Poverty line (US$)** | **Change in impoverishment headcount (%)** | **Standard error** | **T-statistic** |
| --- | --- | --- | --- |
| 1.90 | 0.1292 | 0.1693 | 0.7632 |
| 1.91 | 0.1105 | 0.1485 | 0.7438 |
| 1.92 | 0.1269 | 0.1304 | 0.9739 |
| 1.93 | 0.1286 | 0.1298 | 0.9904 |
| 1.94 | 0.1199 | 0.1333 | 0.8996 |
| 1.95 | 0.1223 | 0.1340 | 0.9133 |
| 1.96 | 0.0936 | 0.1347 | 0.6949 |
| 1.97 | 0.1663 | 0.1284 | 1.2949 |
| 1.98 | 0.2153 | 0.1258 | 1.7115 |
| 1.99 | 0.1721 | 0.1341 | 1.2828 |
| 2.00 | 0.2142 | 0.1507 | 1.4208 |
| 2.01 | 0.1586 | 0.1185 | 1.3380 |
| 2.02 | 0.1638 | 0.1115 | 1.4697 |
| 2.03 | 0.1288 | 0.1101 | 1.1699 |
| 2.04 | 0.1023 | 0.1155 | 0.8853 |
| 2.05 | 0.0022 | 0.1176 | 0.0183 |
| 2.06 | -0.0368 | 0.0905 | -0.4067 |
| 2.07 | 0.0290 | 0.0848 | 0.3419 |
| 2.08 | 0.0277 | 0.0846 | 0.3276 |
| 2.09 | 0.0538 | 0.0867 | 0.6209 |
| 2.10 | 0.0993 | 0.1006 | 0.9869 |
| 2.11 | 0.1311 | 0.0968 | 1.3538 |
| 2.12 | 0.1245 | 0.0942 | 1.3221 |
| 2.13 | 0.0233 | 0.1305 | 0.1786 |
| 2.14 | 0.0950 | 0.1140 | 0.8329 |
| 2.15 | 0.0418 | 0.1088 | 0.3838 |
| 2.16 | 0.0128 | 0.0826 | 0.1549 |
| 2.17 | 0.1073 | 0.1062 | 1.0104 |
| 2.18 | 0.0829 | 0.0967 | 0.8577 |
| 2.19 | 0.0184 | 0.0567 | 0.3251 |
| 2.20 | -0.0012 | 0.0534 | -0.0231 |
| 2.21 | 0.0224 | 0.0540 | 0.4152 |
| 2.22 | -0.0038 | 0.0598 | -0.0629 |
| 2.23 | 0.0273 | 0.0517 | 0.5278 |
| 2.24 | 0.0679 | 0.0540 | 1.2574 |
| 2.25 | 0.0585 | 0.0471 | 1.2414 |
| 2.26 | 0.0551 | 0.0475 | 1.1605 |
| 2.27 | 0.1025 | 0.0871 | 1.1769 |
| 2.28 | 0.0415 | 0.0386 | 1.0748 |
| 2.29 | 0.0419 | 0.0346 | 1.2121 |
| 2.30 | 0.1081 | 0.0917 | 1.1787 |
| 2.31 | 0.0880 | 0.0919 | 0.9578 |
| 2.32 | 0.1267 | 0.0957 | 1.3231 |
| 2.33 | 0.1210 | 0.0960 | 1.2601 |
| 2.34 | 0.1639 | 0.0951 | 1.7235 |
| 2.35 | 0.0898 | 0.0571 | 1.5730 |
| 2.36 | 0.1066 | 0.0520 | 2.0496 |
| 2.37 | 0.1338 | 0.0865 | 1.5463 |
| 2.38 | 0.0921 | 0.0819 | 1.1242 |
| 2.39 | 0.1258 | 0.0961 | 1.3101 |
| 2.40 | 0.1774 | 0.1095 | 1.6198 |
| 2.41 | 0.2275 | 0.1184 | 1.9220 |
| 2.42 | 0.1405 | 0.0956 | 1.4689 |
| 2.43 | 0.1584 | 0.0940 | 1.6838 |
| 2.44 | 0.1051 | 0.0795 | 1.3223 |
| 2.45 | 0.0210 | 0.0974 | 0.2155 |
| 2.46 | 0.0156 | 0.0975 | 0.1601 |
| 2.47 | 0.0845 | 0.0954 | 0.8860 |
| 2.48 | 0.1128 | 0.0969 | 1.1647 |
| 2.49 | 0.1859 | 0.0845 | 2.1991 |
| 2.50 | 0.1227 | 0.0751 | 1.6340 |
| 2.51 | 0.1225 | 0.0756 | 1.6211 |
| 2.52 | 0.0979 | 0.0694 | 1.4110 |
| 2.53 | 0.0611 | 0.0654 | 0.9346 |
| 2.54 | 0.0417 | 0.0687 | 0.6070 |
| 2.55 | 0.0765 | 0.0692 | 1.1058 |
| 2.56 | 0.0648 | 0.0652 | 0.9936 |
| 2.57 | 0.1006 | 0.0617 | 1.6304 |
| 2.58 | 0.0892 | 0.0607 | 1.4701 |
| 2.59 | 0.1295 | 0.0720 | 1.7994 |
| 2.60 | 0.1168 | 0.0568 | 2.0562 |
| 2.61 | 0.0417 | 0.0315 | 1.3239 |
| 2.62 | 0.0749 | 0.0492 | 1.5208 |
| 2.63 | 0.0755 | 0.0489 | 1.5448 |
| 2.64 | 0.0427 | 0.0314 | 1.3614 |
| 2.65 | 0.0908 | 0.0466 | 1.9483 |
| 2.66 | 0.0399 | 0.0462 | 0.8627 |
| 2.67 | 0.0345 | 0.0467 | 0.7378 |
| 2.68 | 0.0260 | 0.0454 | 0.5724 |
| 2.69 | 0.0271 | 0.0459 | 0.5913 |
| 2.70 | 0.0547 | 0.0481 | 1.1372 |
| 2.71 | 0.0498 | 0.0472 | 1.0533 |
| 2.72 | 0.0456 | 0.0351 | 1.2990 |
| 2.73 | 0.0410 | 0.0328 | 1.2494 |
| 2.74 | 0.0266 | 0.0282 | 0.9404 |
| 2.75 | 0.0164 | 0.0270 | 0.6065 |
| 2.76 | 0.0336 | 0.0307 | 1.0960 |
| 2.77 | 0.0711 | 0.0520 | 1.3667 |
| 2.78 | 0.0803 | 0.0533 | 1.5078 |
| 2.79 | 0.0033 | 0.0805 | 0.0406 |
| 2.80 | 0.0451 | 0.0571 | 0.7887 |
| 2.81 | 0.0445 | 0.0574 | 0.7763 |
| 2.82 | 0.0237 | 0.0368 | 0.6422 |
| 2.83 | 0.0015 | 0.0450 | 0.0343 |
| 2.84 | 0.1390 | 0.1146 | 1.2133 |
| 2.85 | -0.0147 | 0.0267 | -0.5512 |
| 2.86 | 0.0402 | 0.0278 | 1.4440 |
| 2.87 | 0.1080 | 0.0727 | 1.4859 |
| 2.88 | 0.1165 | 0.0736 | 1.5823 |
| 2.89 | 0.0770 | 0.0734 | 1.0501 |
| 2.90 | 0.0867 | 0.0736 | 1.1777 |
| 2.91 | 0.1602 | 0.1037 | 1.5439 |
| 2.92 | -0.0107 | 0.0344 | -0.3121 |
| 2.93 | 0.0099 | 0.0399 | 0.2472 |
| 2.94 | 0.0592 | 0.0337 | 1.7574 |
| 2.95 | 0.0139 | 0.0406 | 0.3435 |
| 2.96 | 0.0149 | 0.0399 | 0.3727 |
| 2.97 | 0.0236 | 0.0327 | 0.7219 |
| 2.98 | 0.0250 | 0.0336 | 0.7439 |
| 2.99 | 0.0091 | 0.0302 | 0.3025 |
| 3.00 | 0.0063 | 0.0209 | 0.3029 |
| 3.01 | 0.0053 | 0.0345 | 0.1524 |
| 3.02 | 0.0095 | 0.0343 | 0.2760 |
| 3.03 | 0.0057 | 0.0314 | 0.1826 |
| 3.04 | 0.0175 | 0.0257 | 0.6815 |
| 3.05 | 0.0244 | 0.0269 | 0.9055 |
| 3.06 | 0.0420 | 0.0306 | 1.3713 |
| 3.07 | 0.0377 | 0.0373 | 1.0092 |
| 3.08 | 0.0458 | 0.0360 | 1.2734 |
| 3.09 | -0.0014 | 0.0392 | -0.0364 |
| 3.10 | 0.0028 | 0.0344 | 0.0827 |

*Note*: The results in this Table are shown using poverty lines with an interval of $0.01. However, the corresponding Figure in the main manuscript uses a detailed set of poverty lines with a narrower interval.

Table 4: The effect of poverty lines on the assessment of impoverishment headcount from out-of-pocket health spending between north west and north east regions in Nigeria, 2008/09

| **Poverty line (US$)** | **Change in impoverishment headcount (%)** | **Standard error** | **T-statistic** |
| --- | --- | --- | --- |
| 1.90 | 0.0050 | 0.1260 | 0.0395 |
| 1.91 | -0.0635 | 0.1287 | -0.4932 |
| 1.92 | -0.0676 | 0.1019 | -0.6638 |
| 1.93 | 0.0308 | 0.0706 | 0.4359 |
| 1.94 | 0.1017 | 0.0689 | 1.4766 |
| 1.95 | 0.0335 | 0.0781 | 0.4281 |
| 1.96 | 0.0550 | 0.0795 | 0.6916 |
| 1.97 | -0.0022 | 0.0689 | -0.0324 |
| 1.98 | -0.0926 | 0.0706 | -1.3115 |
| 1.99 | 0.0448 | 0.0615 | 0.7287 |
| 2.00 | 0.0675 | 0.0630 | 1.0719 |
| 2.01 | -0.0275 | 0.0787 | -0.3490 |
| 2.02 | -0.0661 | 0.0781 | -0.8462 |
| 2.03 | -0.0776 | 0.0807 | -0.9625 |
| 2.04 | -0.0200 | 0.0858 | -0.2327 |
| 2.05 | -0.0206 | 0.1061 | -0.1938 |
| 2.06 | -0.0145 | 0.0924 | -0.1569 |
| 2.07 | -0.0215 | 0.0812 | -0.2645 |
| 2.08 | -0.0593 | 0.0851 | -0.6967 |
| 2.09 | -0.0226 | 0.0670 | -0.3377 |
| 2.10 | -0.0421 | 0.0736 | -0.5724 |
| 2.11 | -0.0445 | 0.0791 | -0.5622 |
| 2.12 | -0.0476 | 0.0659 | -0.7223 |
| 2.13 | 0.0881 | 0.1020 | 0.8636 |
| 2.14 | -0.0135 | 0.0793 | -0.1706 |
| 2.15 | 0.0342 | 0.0747 | 0.4578 |
| 2.16 | 0.1094 | 0.0557 | 1.9617 |
| 2.17 | 0.0448 | 0.0446 | 1.0049 |
| 2.18 | 0.0455 | 0.0391 | 1.1636 |
| 2.19 | 0.0034 | 0.0482 | 0.0701 |
| 2.20 | -0.0277 | 0.0562 | -0.4934 |
| 2.21 | -0.0305 | 0.0549 | -0.5560 |
| 2.22 | 0.0193 | 0.0494 | 0.3914 |
| 2.23 | -0.0207 | 0.0398 | -0.5200 |
| 2.24 | -0.0126 | 0.0364 | -0.3464 |
| 2.25 | -0.0370 | 0.0340 | -1.0864 |
| 2.26 | -0.0216 | 0.0336 | -0.6447 |
| 2.27 | -0.0199 | 0.0368 | -0.5421 |
| 2.28 | -0.0309 | 0.0411 | -0.7502 |
| 2.29 | -0.0556 | 0.0447 | -1.2450 |
| 2.30 | -0.0528 | 0.0447 | -1.1823 |
| 2.31 | -0.0471 | 0.0464 | -1.0143 |
| 2.32 | -0.0219 | 0.0396 | -0.5517 |
| 2.33 | -0.0355 | 0.0463 | -0.7667 |
| 2.34 | -0.0588 | 0.0401 | -1.4660 |
| 2.35 | -0.0231 | 0.0407 | -0.5681 |
| 2.36 | -0.0701 | 0.0435 | -1.6139 |
| 2.37 | -0.0327 | 0.0407 | -0.8034 |
| 2.38 | -0.0080 | 0.0441 | -0.1807 |
| 2.39 | -0.0262 | 0.0530 | -0.4944 |
| 2.40 | -0.0326 | 0.0528 | -0.6172 |
| 2.41 | 0.0392 | 0.0419 | 0.9363 |
| 2.42 | 0.0392 | 0.0471 | 0.8321 |
| 2.43 | 0.0617 | 0.0334 | 1.8464 |
| 2.44 | 0.0466 | 0.0345 | 1.3491 |
| 2.45 | 0.1190 | 0.0682 | 1.7448 |
| 2.46 | 0.0727 | 0.0763 | 0.9531 |
| 2.47 | 0.0049 | 0.0727 | 0.0678 |
| 2.48 | 0.0464 | 0.0682 | 0.6793 |
| 2.49 | -0.0122 | 0.0435 | -0.2797 |
| 2.50 | 0.0122 | 0.0434 | 0.2816 |
| 2.51 | 0.0176 | 0.0449 | 0.3933 |
| 2.52 | 0.0145 | 0.0463 | 0.3124 |
| 2.53 | 0.0272 | 0.0458 | 0.5951 |
| 2.54 | 0.0674 | 0.0396 | 1.7008 |
| 2.55 | 0.0497 | 0.0374 | 1.3294 |
| 2.56 | 0.0369 | 0.0399 | 0.9243 |
| 2.57 | 0.0203 | 0.0317 | 0.6401 |
| 2.58 | 0.0178 | 0.0331 | 0.5389 |
| 2.59 | 0.0142 | 0.0346 | 0.4105 |
| 2.60 | 0.0033 | 0.0219 | 0.1506 |
| 2.61 | -0.0235 | 0.0231 | -1.0202 |
| 2.62 | -0.0127 | 0.0231 | -0.5514 |
| 2.63 | -0.0421 | 0.0369 | -1.1387 |
| 2.64 | -0.0648 | 0.0387 | -1.6743 |
| 2.65 | -0.0134 | 0.0236 | -0.5663 |
| 2.66 | 0.0011 | 0.0433 | 0.0243 |
| 2.67 | 0.0298 | 0.0358 | 0.8322 |
| 2.68 | 0.0347 | 0.0345 | 1.0045 |
| 2.69 | 0.0389 | 0.0348 | 1.1189 |
| 2.70 | 0.0235 | 0.0277 | 0.8489 |
| 2.71 | 0.0204 | 0.0278 | 0.7358 |
| 2.72 | -0.0204 | 0.0277 | -0.7356 |
| 2.73 | -0.0322 | 0.0277 | -1.1628 |
| 2.74 | -0.0157 | 0.0236 | -0.6656 |
| 2.75 | -0.0172 | 0.0264 | -0.6501 |
| 2.76 | 0.0087 | 0.0210 | 0.4121 |
| 2.77 | 0.0140 | 0.0234 | 0.5974 |
| 2.78 | 0.0133 | 0.0227 | 0.5887 |
| 2.79 | 0.0792 | 0.0659 | 1.2020 |
| 2.80 | 0.0097 | 0.0342 | 0.2841 |
| 2.81 | 0.0351 | 0.0275 | 1.2735 |
| 2.82 | 0.0129 | 0.0249 | 0.5159 |
| 2.83 | 0.0383 | 0.0348 | 1.1002 |
| 2.84 | -0.0181 | 0.0373 | -0.4853 |
| 2.85 | 0.0251 | 0.0267 | 0.9426 |
| 2.86 | -0.0016 | 0.0179 | -0.0904 |
| 2.87 | 0.0260 | 0.0142 | 1.8288 |
| 2.88 | -0.0076 | 0.0370 | -0.2066 |
| 2.89 | 0.0094 | 0.0427 | 0.2196 |
| 2.90 | -0.0230 | 0.0462 | -0.4981 |
| 2.91 | -0.0243 | 0.0466 | -0.5224 |
| 2.92 | -0.0117 | 0.0370 | -0.3167 |
| 2.93 | -0.0012 | 0.0371 | -0.0324 |
| 2.94 | -0.0545 | 0.0279 | -1.9521 |
| 2.95 | -0.0150 | 0.0364 | -0.4135 |
| 2.96 | 0.0004 | 0.0335 | 0.0121 |
| 2.97 | -0.0150 | 0.0323 | -0.4651 |
| 2.98 | -0.0050 | 0.0318 | -0.1576 |
| 2.99 | -0.0101 | 0.0321 | -0.3161 |
| 3.00 | -0.0348 | 0.0274 | -1.2693 |
| 3.01 | 0.0046 | 0.0306 | 0.1498 |
| 3.02 | 0.0102 | 0.0282 | 0.3623 |
| 3.03 | 0.0048 | 0.0272 | 0.1762 |
| 3.04 | -0.0086 | 0.0207 | -0.4124 |
| 3.05 | -0.0264 | 0.0302 | -0.8728 |
| 3.06 | -0.0023 | 0.0206 | -0.1109 |
| 3.07 | 0.0144 | 0.0277 | 0.5222 |
| 3.08 | -0.0071 | 0.0280 | -0.2543 |
| 3.09 | 0.0452 | 0.0329 | 1.3772 |
| 3.10 | 0.0373 | 0.0271 | 1.3780 |

*Note*: The results in this Table are shown using poverty lines with an interval of $0.01. However, the corresponding Figure in the main manuscript uses a detailed set of poverty lines with a narrower interval.

Table 5: The effect of poverty lines on the assessment of impoverishment headcount from out-of-pocket health spending between south west and south east regions in Nigeria, 2008/09

| **Poverty line (US$)** | **Change in impoverishment headcount (%)** | **Standard error** | **T-statistic** |
| --- | --- | --- | --- |
| 1.90 | 0.2035 | 0.2271 | 0.8962 |
| 1.91 | 0.1285 | 0.2399 | 0.5355 |
| 1.92 | 0.0054 | 0.2300 | 0.0236 |
| 1.93 | -0.0352 | 0.2150 | -0.1639 |
| 1.94 | 0.0778 | 0.2092 | 0.3720 |
| 1.95 | 0.1441 | 0.2090 | 0.6896 |
| 1.96 | 0.1649 | 0.2057 | 0.8015 |
| 1.97 | 0.1744 | 0.2033 | 0.8580 |
| 1.98 | 0.2261 | 0.2067 | 1.0937 |
| 1.99 | 0.2641 | 0.2078 | 1.2711 |
| 2.00 | 0.2175 | 0.2016 | 1.0788 |
| 2.01 | 0.1005 | 0.1924 | 0.5223 |
| 2.02 | -0.0542 | 0.1951 | -0.2777 |
| 2.03 | -0.3120 | 0.1388 | -2.2477 |
| 2.04 | -0.2732 | 0.0979 | -2.7902 |
| 2.05 | -0.1311 | 0.2461 | -0.5325 |
| 2.06 | -0.4284 | 0.1670 | -2.5651 |
| 2.07 | -0.2899 | 0.1657 | -1.7490 |
| 2.08 | -0.4781 | 0.2450 | -1.9515 |
| 2.09 | -0.2049 | 0.2049 | -1.0000 |
| 2.10 | -0.1092 | 0.1015 | -1.0756 |
| 2.11 | 0.0094 | 0.1391 | 0.0676 |
| 2.12 | -0.0748 | 0.1261 | -0.5933 |
| 2.13 | -0.0391 | 0.1321 | -0.2958 |
| 2.14 | -0.0139 | 0.1208 | -0.1149 |
| 2.15 | -0.1101 | 0.0972 | -1.1326 |
| 2.16 | -0.1425 | 0.1034 | -1.3785 |
| 2.17 | -0.0509 | 0.0831 | -0.6122 |
| 2.18 | -0.0596 | 0.0823 | -0.7247 |
| 2.19 | -0.0803 | 0.0684 | -1.1743 |
| 2.20 | -0.1655 | 0.0818 | -2.0223 |
| 2.21 | -0.1769 | 0.0910 | -1.9442 |
| 2.22 | -0.1611 | 0.0703 | -2.2926 |
| 2.23 | -0.1103 | 0.0707 | -1.5598 |
| 2.24 | -0.0566 | 0.0761 | -0.7429 |
| 2.25 | -0.1229 | 0.0637 | -1.9299 |
| 2.26 | -0.0143 | 0.0909 | -0.1578 |
| 2.27 | -0.0185 | 0.1038 | -0.1781 |
| 2.28 | -0.0618 | 0.1644 | -0.3761 |
| 2.29 | -0.0984 | 0.1622 | -0.6063 |
| 2.30 | 0.0402 | 0.1819 | 0.2209 |
| 2.31 | -0.1148 | 0.1992 | -0.5761 |
| 2.32 | -0.1437 | 0.1990 | -0.7222 |
| 2.33 | -0.1396 | 0.1958 | -0.7129 |
| 2.34 | -0.2716 | 0.1790 | -1.5172 |
| 2.35 | -0.2167 | 0.2000 | -1.0833 |
| 2.36 | -0.0367 | 0.1595 | -0.2298 |
| 2.37 | -0.0851 | 0.1309 | -0.6504 |
| 2.38 | -0.0359 | 0.1077 | -0.3331 |
| 2.39 | -0.1452 | 0.0963 | -1.5070 |
| 2.40 | -0.0975 | 0.1088 | -0.8962 |
| 2.41 | 0.0640 | 0.0806 | 0.7949 |
| 2.42 | 0.0815 | 0.0869 | 0.9380 |
| 2.43 | 0.0335 | 0.0839 | 0.3996 |
| 2.44 | 0.0180 | 0.0871 | 0.2071 |
| 2.45 | 0.1456 | 0.0869 | 1.6749 |
| 2.46 | 0.2053 | 0.1268 | 1.6192 |
| 2.47 | 0.0972 | 0.1084 | 0.8970 |
| 2.48 | 0.0327 | 0.1031 | 0.3171 |
| 2.49 | 0.0456 | 0.1290 | 0.3530 |
| 2.50 | 0.0436 | 0.1109 | 0.3930 |
| 2.51 | -0.0120 | 0.0598 | -0.1998 |
| 2.52 | -0.0194 | 0.0627 | -0.3095 |
| 2.53 | -0.0756 | 0.0684 | -1.1053 |
| 2.54 | -0.0416 | 0.0653 | -0.6360 |
| 2.55 | 0.0188 | 0.0653 | 0.2878 |
| 2.56 | -0.0152 | 0.0598 | -0.2552 |
| 2.57 | -0.0097 | 0.0695 | -0.1392 |
| 2.58 | -0.0123 | 0.0776 | -0.1582 |
| 2.59 | 0.0048 | 0.0790 | 0.0604 |
| 2.60 | 0.0350 | 0.1090 | 0.3215 |
| 2.61 | -0.0380 | 0.1178 | -0.3224 |
| 2.62 | -0.0294 | 0.0973 | -0.3018 |
| 2.63 | -0.0851 | 0.0774 | -1.0991 |
| 2.64 | -0.0969 | 0.0821 | -1.1802 |
| 2.65 | -0.1599 | 0.1115 | -1.4347 |
| 2.66 | -0.1116 | 0.0789 | -1.4147 |
| 2.67 | -0.1389 | 0.0789 | -1.7612 |
| 2.68 | 0.1747 | 0.2374 | 0.7356 |
| 2.69 | 0.2209 | 0.2369 | 0.9324 |
| 2.70 | 0.2439 | 0.2380 | 1.0245 |
| 2.71 | 0.1937 | 0.2368 | 0.8179 |
| 2.72 | -0.0695 | 0.0749 | -0.9280 |
| 2.73 | -0.0545 | 0.0802 | -0.6792 |
| 2.74 | -0.1045 | 0.1158 | -0.9027 |
| 2.75 | -0.0893 | 0.1131 | -0.7890 |
| 2.76 | -0.0319 | 0.0770 | -0.4142 |
| 2.77 | -0.0711 | 0.0784 | -0.9062 |
| 2.78 | -0.0895 | 0.0735 | -1.2169 |
| 2.79 | -0.1077 | 0.0793 | -1.3584 |
| 2.80 | -0.0615 | 0.0891 | -0.6909 |
| 2.81 | -0.0738 | 0.0918 | -0.8040 |
| 2.82 | -0.1627 | 0.1071 | -1.5185 |
| 2.83 | -0.1554 | 0.0990 | -1.5695 |
| 2.84 | -0.0952 | 0.0915 | -1.0399 |
| 2.85 | 0.0532 | 0.0972 | 0.5470 |
| 2.86 | 0.0106 | 0.0901 | 0.1180 |
| 2.87 | -0.0513 | 0.0847 | -0.6060 |
| 2.88 | -0.0650 | 0.0850 | -0.7645 |
| 2.89 | -0.0724 | 0.1388 | -0.5217 |
| 2.90 | -0.0830 | 0.1262 | -0.6576 |
| 2.91 | 0.0589 | 0.0728 | 0.8089 |
| 2.92 | 0.0470 | 0.0737 | 0.6373 |
| 2.93 | 0.0593 | 0.0718 | 0.8263 |
| 2.94 | 0.0664 | 0.0745 | 0.8916 |
| 2.95 | -0.0040 | 0.1026 | -0.0394 |
| 2.96 | -0.0456 | 0.1061 | -0.4297 |
| 2.97 | -0.1303 | 0.0920 | -1.4166 |
| 2.98 | -0.0815 | 0.1105 | -0.7372 |
| 2.99 | -0.1199 | 0.0694 | -1.7263 |
| 3.00 | -0.0516 | 0.0954 | -0.5406 |
| 3.01 | 0.0482 | 0.0959 | 0.5028 |
| 3.02 | -0.0473 | 0.0730 | -0.6480 |
| 3.03 | -0.1192 | 0.0791 | -1.5061 |
| 3.04 | -0.2052 | 0.1235 | -1.6616 |
| 3.05 | -0.1700 | 0.1358 | -1.2524 |
| 3.06 | -0.1365 | 0.1366 | -0.9990 |
| 3.07 | -0.0443 | 0.0985 | -0.4502 |
| 3.08 | -0.1357 | 0.0758 | -1.7900 |
| 3.09 | -0.1076 | 0.0754 | -1.4263 |
| 3.10 | -0.1273 | 0.0721 | -1.7654 |

*Note*: The results in this Table are shown using poverty lines with an interval of $0.01. However, the corresponding Figure in the main manuscript uses a detailed set of poverty lines with a narrower interval.
